# Neural processing of visual information under interocular suppression: a critical review

**DOI:** 10.3389/fpsyg.2014.00453

**Published:** 2014-05-19

**Authors:** Philipp Sterzer, Timo Stein, Karin Ludwig, Marcus Rothkirch, Guido Hesselmann

**Affiliations:** ^1^Visual Perception Laboratory, Department of Psychiatry and Psychotherapy, Charité UniversitätsmedizinBerlin, Germany; ^2^Center for Mind/Brain Sciences, University of TrentoRovereto, Italy; ^3^Department of Psychology, Humboldt-Universität zu BerlinBerlin, Germany

**Keywords:** visual perception, binocular rivalry, interocular suppression, neuroimaging, consciousness

## Abstract

When dissimilar stimuli are presented to the two eyes, only one stimulus dominates at a time while the other stimulus is invisible due to interocular suppression. When both stimuli are equally potent in competing for awareness, perception alternates spontaneously between the two stimuli, a phenomenon called binocular rivalry. However, when one stimulus is much stronger, e.g., due to higher contrast, the weaker stimulus can be suppressed for prolonged periods of time. A technique that has recently become very popular for the investigation of unconscious visual processing is continuous flash suppression (CFS): High-contrast dynamic patterns shown to one eye can render a low-contrast stimulus shown to the other eye invisible for up to minutes. Studies using CFS have produced new insights but also controversies regarding the types of visual information that can be processed unconsciously as well as the neural sites and the relevance of such unconscious processing. Here, we review the current state of knowledge in regard to neural processing of interocularly suppressed information. Focusing on recent neuroimaging findings, we discuss whether and to what degree such suppressed visual information is processed at early and more advanced levels of the visual processing hierarchy. We review controversial findings related to the influence of attention on early visual processing under interocular suppression, the putative differential roles of dorsal and ventral areas in unconscious object processing, and evidence suggesting privileged unconscious processing of emotional and other socially relevant information. On a more general note, we discuss methodological and conceptual issues, from practical issues of how unawareness of a stimulus is assessed to the overarching question of what constitutes an adequate operational definition of unawareness. Finally, we propose approaches for future research to resolve current controversies in this exciting research area.

## INTRODUCTION

When two conflicting images are presented to the two eyes, they usually do not merge into a mixture, but rather tend to rival for exclusive perceptual dominance. When both stimuli are equally potent in competing for dominance, such binocular rivalry typically results in perceptual alternations between the two images every few seconds, similar to other bistable perceptual phenomena that occur during viewing of ambiguous visual stimuli such as the Necker cube or ambiguous motion stimuli (Blake and Logothetis, 2002; Sterzer et al., 2009b). Whenever one of the two rivaling images dominates conscious perception, the other respective image is suppressed from conscious awareness for several seconds. This interocular suppression of visual stimuli through binocular rivalry offers a unique opportunity to study neural responses to visual stimuli in the absence of conscious awareness. However, the assessment of awareness during binocular rivalry in its traditional form is complicated by the fact that it relies entirely on the observers’ reports about their subjective visual experience. Moreover, dominance of one image and suppression of the other image are not always complete (*piecemeal rivalry*) and transitions between perceptual states occur largely stochastically and are thus unpredictable to both the observer and the experimenter (Blake and Logothetis, 2002). It is therefore, on the basis of subjective reports of perceptual states during conventional binocular rivalry, difficult to reliably determine at which time exactly an image is suppressed and whether it is fully suppressed from awareness.

One variant of binocular rivalry that allows the experimenter to control perceptual dominance at least for brief periods of time is a technique called *flash suppression* (Wolfe, 1984): when one of the two rivaling images is presented first monocularly, followed by binocular presentation of the two images, the image presented first is likely to be suppressed from awareness at the beginning of binocular presentation. A further modification of this technique, *continuous flash suppression* (CFS), can be used to reliably suppress an image for several seconds or even minutes. For CFS, dynamic high-contrast Mondrian-like patterns (also referred to as CFS masks) are flashed to one eye, rendering lower-contrast stimuli presented to the other eye invisible for extended periods of time (Tsuchiya and Koch, 2005). It should be mentioned that it is not yet clear whether CFS should be regarded as a variant of binocular rivalry that induces particularly strong suppression (Shimaoka and Kaneko, 2011), or whether CFS is supported by mechanisms distinct from binocular rivalry (Tsuchiya et al., 2006).

In the following, we critically review research that examined the neural fate of stimulus information that is suppressed from awareness through interocular suppression, with a focus on the neuroimaging literature. In the first section of this article, we discuss methodological problems in the neuroscientific study of unconscious information processing that pose challenges for the interpretation of the neural signals measured in response to suppressed visual stimuli. The second section reviews studies that investigated the processing of suppressed stimuli in early visual cortex and, in particular, the relationship of awareness and attention in early visual processing. The final part of this article is concerned with the processing of suppressed stimuli in higher-level visual areas, highlighting a recent controversy in regard to dissociable roles of ventral and dorsal stream areas in unconscious information processing. We will close by pointing out possible approaches that we think might help to tackle the methodological problems and heterogeneity of findings in future research.

## OBJECTIVE VS. SUBJECTIVE MEASURES OF (UN-)AWARENESS

When conducting research on the neural correlates of visual information processing outside awareness, the experimenter has no direct access to the participant’s subjective visual experience of the presented stimuli (Malach, 2008; Seth et al., 2008). Thus, the desired correlation between specific conscious contents (e.g., stimulus seen or not seen) and neuronal activity cannot be directly measured. Ultimately, only correlations between behavioral indications of conscious contents (e.g., verbal reports, button presses) and measures of brain states [e.g., functional magnetic resonance imaging (fMRI), electroencephalography (EEG), magnetoencephalography (MEG)] can be investigated (Overgaard, 2006). As a consequence, activity related to conscious contents needs to be disentangled from response-related and all other unrelated neuronal activity (Aru et al., 2012; de Graaf et al., 2012; Frassle et al., 2014). At a more fundamental level, the question of which type of behavioral report classifies as a valid measure of awareness needs to be answered. Not surprisingly, the debate on the optimal measure of conscious vs. unconscious perception has been a long-standing one in the cognitive sciences (Reingold and Merikle, 1990; Merikle and Reingold, 1998; Kunimoto et al., 2001). An important conceptual and methodological aspect of studies investigating visual processing under interocular suppression concerns the assessment of *un*awareness of a stimulus (Snodgrass, 2004; Pessoa, 2005). The fundamental problem with observers’ introspective reports regarding their unawareness of a stimulus is that their report critically depends on subjective criteria. Accordingly, the major criticism of introspection has been that subjective reports are generally susceptible to influences of response bias (Eriksen, 1960; Holender, 1986). Especially in the face of weak or noisy signals, observers might show systematically low confidence in a visual discrimination task, which could be falsely interpreted as an absence of awareness even though a trace of awareness was present but not reported (Bjorkman et al., 1993).

In contrast to such *subjective* measures of unawareness, an observer can be regarded as *objectively* unaware when performance in a “forced-choice” task is at chance level. For instance, when participants have to report in which of two successive intervals a target stimulus was presented, or whether the stimulus belonged to category A or B, above chance level performance indicates awareness of the stimulus, whereas performance not significantly different from chance level indicates the absence of awareness. In the examples given above, chance level in the two-alternative forced-choice tasks would be expressed as 50%, or as *d*′ = 0, with *d*′ representing the perceptual sensitivity measure within the mathematical framework of signal detection theory widely used in psychophysics (Green and Swets, 1966; Macmillan and Creelman, 1991). A challenge to the purely objective criterion is the conceivable situation in which participants perform above chance in one task, whereas their performance is at chance level in another task related to the same stimulus. For instance, observers can be at chance level in discriminating the orientation of a pattern while being significantly above chance level in discriminating its location (Zadbood et al., 2011; see also Hong and Blake, 2009). When measuring neural signals associated with the presentation of stimuli outside awareness, it is thus important to precisely define which aspects of the stimuli observers are unaware of. Here we argue that chance level performance has to be demonstrated for the same discrimination that constitutes the dimension of interest in concurrent brain activity recordings. For example, when brain responses to supposedly invisible fearful vs. neutral faces are recorded, participants should be at chance in discriminating between fearful and neutral faces (and not in discriminating between, e.g., intact and scrambled faces).

A critical point concerning objective measures of unawareness is the statistical method that is used to prove that performance is “at chance level.” For the objective criterion, one needs to assure that the null hypothesis is true. In this case classical statistics – which test how likely it is for the observed data to occur if the null hypothesis were true – are insufficient (Merikle and Daneman, 2000; Schmidt and Vorberg, 2006). If testing the data against 0, using for example a *t*-test, a *p*-value smaller than 0.05 implies that the null hypothesis can be rejected with an error probability smaller than 5%. However, a *p*-value >0.05 does *not* imply that the null hypothesis is true. In that case the test just has no result, that is, the evidence is not sufficient to support a conclusion (Dienes, 2011). Other statistical methods are therefore needed when our goal is to state evidence for the null hypothesis, which is the case when we want to establish chance-level performance as a proof of objective unawareness. Possible solutions are the use of power analyses (Faul et al., 2007), equivalence confidence intervals (Berger and Hsu, 1996; Overgaard et al., 2013), or Bayesian statistics. In Bayesian statistics, the posterior probability of a hypothesis is tested conditional on the observed data and a prior probability. It is thus possible to directly test two hypotheses against each other and – more importantly – compute a probability value for each of these hypotheses, also if one of them is the null hypothesis. For Bayesian statistics, the two hypotheses need to be defined in terms of prior probability distributions, or “priors.” The null hypothesis can be defined as a Dirac delta function, i.e., a function in which every *x*-value is 0 except at 0. The alternative hypothesis should be modeled according to prior empirical or theoretical knowledge, e.g., as a uniform, normal or half-normal distribution (Dienes, in press). The upper and lower bound or the mean and standard deviation of the respective distribution can be inferred from, e.g., a supraliminal experimental condition or previous research. In order to evaluate chance performance individually for each participant, Rouder et al. (2007) suggest to use a “mass-at-chance model,” which is based on Bayesian analyses and gives an – albeit conservative – estimation of the probability that a participant’s performance is truly at chance level. Irrespective of the application of this model, one of its virtues is that it demonstrates the importance of having enough power for claims of chance performance.

The assessment of unawareness on the basis of objective criteria alone may be overly conservative as it disregards the observer’s introspective account and may overestimate conscious perception in cases where forced-choice tasks are contaminated by unconscious processes. In other words, an observer may be erroneously classified as consciously aware of a stimulus in a situation where motor reports are influenced by some unconscious process, resulting in above chance performance despite phenomenal absence of awareness. Above chance performance in a particular task may thus simply show that stimulus information was processed and had an influence on behavior under conditions in which stimulus processing was not accompanied by awareness (Merikle and Daneman, 2000). Dissociations between introspective reports of visual awareness and objective measures of performance (“blindsight”) are well-known to occur in cortically lesioned patients (Stoerig, 2006) but can also be observed in neurologically intact participants (Meeres and Graves, 1990; Lau and Passingham, 2006; Schwiedrzik et al., 2011). It may thus be helpful to complement the objective assessment of unawareness with the concomitant use of subjective measures, especially because subjective reports can provide a trial-by-trial measure of awareness while objective measures indicate observer’s overall performance in a particular task. One frequently used subjective behavioral report is to let participants directly rate the visibility of the stimulus on a larger (Sergent and Dehaene, 2004) or smaller scale (Ramsoy and Overgaard, 2004). Characteristic of the latter, the 4-point perceptual awareness scale, is its lack of symmetry, because there is only one “invisible” rating as opposed to three different “visible” ratings, ranging from “weak glimpse” and “almost clear” to “absolutely clear.” An alternative and widely applied approach to measure awareness is based on metacognitive (second-order) judgments in the form of confidence ratings. Participants have to indicate their confidence about how accurate their first-order perceptual judgment was (Dienes et al., 1995). For example, participants may be instructed to provide confidence ratings about how well they performed in a preceding stimulus localization task (Rothkirch et al., 2012). Another recently introduced variation on confidence ratings is post-decision wagering, in which confidence levels are expressed in terms of the amount of money the participants are willing bet on their judgments. Presumably, this leads to a higher motivation to reveal all conscious knowledge for the sake of cash rewards (Persaud et al., 2007). However, this approach has also sparked criticism, since wagering behavior is likely biased by subjects’ propensity to avoid losses (Schurger and Sher, 2008; Fleming and Dolan, 2010). Although many current researchers would agree that participants’ introspective phenomenal reports need to be taken seriously by any study of consciousness (Dehaene and Naccache, 2001), the question of which subjective measure is best suited for a given experimental situation remains a matter of ongoing research and debate (Dienes and Seth, 2010; Sandberg et al., 2010; Szczepanowski et al., 2013). One way to overcome the potential confounding factor of response bias is the implementation of signal detection theory in the analysis of subjective reports by calculating a measure of second-order sensitivity (“type-2” *d*′ as opposed to “type-1” *d*′ based on first-order reports), which is independent of response bias or of where participants place their criterion for making high- and low-confidence judgments (Kunimoto et al., 2001; Szczepanowski and Pessoa, 2007; Kanai et al., 2010).

## PROCESSING OF SUPPRESSED VISUAL STIMULI IN EARLY VISUAL CORTEX

**FIGURE 1:**
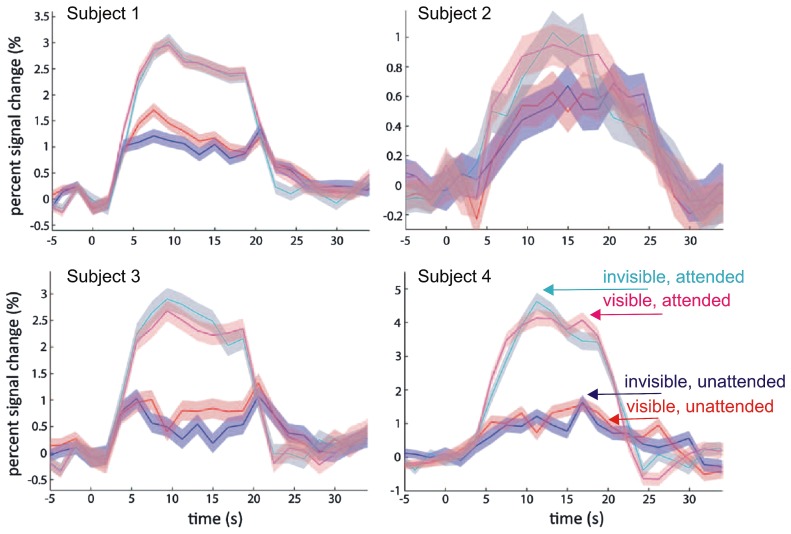
**Modulation of fMRI-BOLD activation in primary visual cortex (V1) by attention and visibility.** Data from Watanabe et al. (2011): Disc-shaped moving target gratings were rendered invisible by non-overlapping CFS masks. Plotted are time courses of averaged BOLD responses (% change) and 95% confidence intervals in the targeted monocular region from four subjects. The data show a modulation by attention (as operationalized by task at fixation), but no modulation by visibility (cyan: invisible attended; magenta: visible attended; blue: invisible unattended; red: visible unattended). Modified with permission from Watanabe et al. (2011; copyright 2011 The American Association for the Advancement of Science).

Human primary visual cortex (V1) constitutes the first cortical processing stage for the largest part of visual signals from the retina. fMRI studies have consistently shown a tight link between stimulus awareness during binocular rivalry and blood-oxygen level dependent (BOLD) activity levels in V1, with invisible stimuli resulting in much reduced activity levels (Polonsky et al., 2000; Tong and Engel, 2001; Haynes et al., 2005; Lee et al., 2005; Wunderlich et al., 2005). However, this set of findings has recently been challenged by a study that aimed to separate the effects of top-down attention and visual awareness on the BOLD signal in human V1 (Watanabe et al., 2011). Based on the notion that attention and awareness are two dissociable processes supporting distinct functions in the visual system (Lamme, 2003; Koch and Tsuchiya, 2007; van Boxtel et al., 2010, but see Cohen et al., 2012), the authors modulated awareness and attention independently of each other in a 2 × 2 factorial design. They used a variant of CFS in which the mask and the target stimulus overlapped only partially, allowing them to isolate target- from mask-related fMRI-BOLD activity in retinotopic V1. Awareness was modulated by presenting mask and target either to the same eye (visible) or to the two eyes separately (invisible). At the same time, visual attention was manipulated by having participants either report the visibility of the target (attended) or perform a demanding letter detection task at fixation (unattended). Replicating a well-established finding from previous work, the authors found stronger target-related V1 responses when the target stimulus was attended in comparison to the unattended condition (Gandhi et al., 1999; Kastner et al., 1999; Martinez et al., 1999), independent of visibility (also see Bahrami et al., 2007, for the effect of attentional load under CFS). In sharp contrast to earlier fMRI results, however, the authors failed to detect stronger V1 activity to visible than to invisible targets (**Figure 1**). Watanabe et al. (2011) concluded that the previously reported awareness modulation on the BOLD signal in V1 might be an artifact caused by the concurrent attentional modulation, and that this could also explain the discrepancy between fMRI studies and single-unit recordings that did not find robust awareness-related changes in firing rates of V1 neurons (Leopold and Logothetis, 1996; Wilke et al., 2006).

**FIGURE 2:**
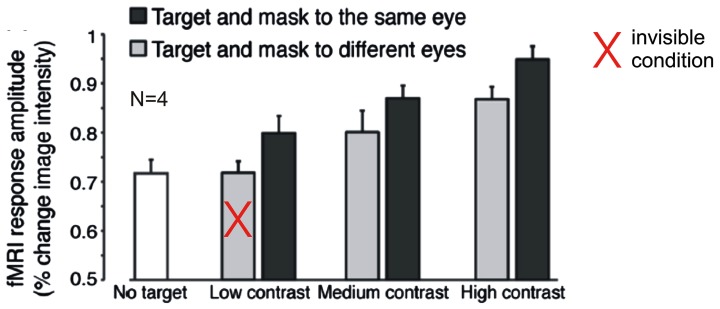
**Modulation of fMRI-BOLD activation in primary visual cortex (V1) by contrast and visibility under inattention.** Data from Yuval-Greenberg and Heeger (2013): Disc-shaped moving target gratings were rendered invisible by non-overlapping CFS masks. Plotted are average BOLD responses (% change) from four subjects. Gray bars indicate trials with target and masks presented to different eyes, black bars indicate trials with target and masks presented to the same eye. The white bar indicates trials with no target (“CFS mask only”). At low target contrast, interocular suppression (different eyes) rendered targets invisible. The data show a modulation by visibility and contrast, even though subjects were engaged in a task at fixation. Modified with permission from Yuval-Greenberg and Heeger (2013; copyright 2013 Society for Neuroscience).

A recently published study casts doubt on this interpretation. Using a very similar stimulus design and attentional manipulation, but with substantially greater statistical power, Yuval-Greenberg and Heeger (2013) did find a significant modulation of target-evoked V1 activity by CFS. When the mask and target were presented dichoptically and the target was suppressed from awareness, V1 activity was at the same level as during presentation of the CFS mask alone; presentation of mask and target to the same eye, however, resulted in target visibility and was associated with significantly greater BOLD activity levels in V1 (**Figure 2**). Interestingly, a similar difference between presentation to same and different eyes of mask and target was also observed for targets with higher contrast that were not fully suppressed from awareness by CFS. The authors concluded that the presence of the CFS mask may suppress neural activity in V1 similar to other forms of visual masking, suggesting that CFS impacts awareness by modulating the gain of neural responses to the target at an early stage of visual processing. Why did Watanabe et al. (2011) in their earlier study using a similar stimulus design fail to find a modulation of V1 BOLD responses by CFS? As Yuval-Greenberg and Heeger (2013) argue, the study by Watanabe et al. (2011) may have been “underpowered,” as they performed only 6–9 experimental runs with just one single trial of 16 s duration per condition in each run, which by current standards in fMRI research is a surprisingly small number of trials indeed. Moreover, the awareness assessment during scanning, in which participants had to distinguish between visible and invisible targets, does not rule out residual visibility even in the “invisible” condition. Participants may have adopted the strategy to label clearly visible targets as visible and less clearly visible targets as invisible, so there is no sufficient proof of target unawareness in the “invisible” condition. (Note that Yuval-Greenberg and Heeger (2013) avoided this issue by including “CFS mask only” trials.) Finally, there were two visible stimulus presentations interspersed in each invisible block (and *vice versa*). These “catch trials” could have attenuated possible BOLD activity differences between visible and invisible blocks.

Given these methodological limitations, the null result regarding V1 activity modulation by CFS reported by Watanabe et al. (2011) cannot be taken as conclusive evidence for the absence of interocular suppression effects in early visual cortex. This conclusion is supported by Yuval-Greenberg and Heeger’s (2013) recent study that provided convincing evidence for a V1 BOLD activity modulation by CFS. However, it is still noteworthy that this modulation is relatively subtle, not only relative to the attention-related modulation observed by Watanabe et al. (2011), but also when compared across studies to the strong CFS-related modulation of BOLD responses to object stimuli in higher-level cortex that are discussed in detail in the next section of this article (Fang and He, 2005; Sterzer et al., 2008; Hesselmann and Malach, 2011; Hesselmann et al., 2011). This observation is relevant for the discrepancy between electrophysiological recordings in monkeys and human fMRI studies that differed in their conclusions regarding the effects of interocular suppression in low-level visual areas (for an in depth discussion see, Tong et al., 2006; Maier et al., 2008). Single unit recordings showed percept-related changes in firing rates in only ~20% of V1/V2 neurons (Leopold and Logothetis, 1996; Keliris et al., 2010). In contrast fMRI studies found much stronger percept-related V1 BOLD signal modulations during binocular rivalry (Polonsky et al., 2000), sometimes even equivalent to those evoke by stimulus changes (Tong and Engel, 2001; Wunderlich et al., 2005, but see Haynes et al., 2005 who found that BOLD signal modulation during rivalry amounted to only 28% of that evoked by stimulus changes). It is indeed possible that the strong rivalry-related BOLD signal modulations reported in these studies are in large part due to concurrent attentional modulation, as suggested by Watanabe et al. (2011). Alternatively, the discrepancy between monkey neurophysiology and human fMRI studies may simply reflect differences in the nature of the measured signals, with V1 spiking activity being less indicative of conscious perception under interocular suppression than V1 low-frequency local-field potentials (Wilke et al., 2006) and V1 BOLD signals (Maier et al., 2008).

## THE FATE OF SUPPRESSED VISUAL INFORMATION BEYOND EARLY VISUAL CORTEX

Early neuroimaging work showed that in high-level extrastriate visual areas the amplitudes of percept-related fMRI signal fluctuations during binocular rivalry are similar to those during actual stimulus alternations (Tong et al., 1998). This finding was initially interpreted as evidence for a resolution of rivalry at early levels through competitive interactions between monocular channels in lateral geniculate nucleus (LGN) and V1, with no maintained representation of the suppressed stimulus at higher levels of the visual processing hierarchy. However, behaviorally the involvement of perceptual (rather than purely interocular) mechanisms is shown by persistence of rivalry when the monocular images are rapidly swapped between the eyes, preventing interocular competition (Logothetis et al., 1996). An influence of perceptual mechanisms is also suggested by the observation that complementary patchworks of intermingled images presented to each eye can drive rivalry (Kovacs et al., 1996). Moreover, binocular rivalry is affected by complex information, such as object category, contained in suppressed stimuli (e.g., Andrews and Blakemore, 1999; Alais and Parker, 2006; see also Blake and Logothetis, 2002; Tong et al., 2006, for reviews), indicating that information from interocularly suppressed stimuli is still processed at sufficiently advanced levels where this information can be extracted and represented.

Recent neuroimaging work has explicitly asked whether complex stimulus information is still represented at advanced stages of the visual processing hierarchy during binocular rivalry suppression, focusing mainly on two questions: first, is visual information that is of special behavioral relevance still processed under interocular suppression? This question is based on the assumption that stimuli of special behavioral relevance, e.g., emotional information (for reviews, see Pessoa, 2005; Vuilleumier, 2005), may undergo preferential and automatic processing in the absence of attention and even awareness. Second, is complex stimulus information such as object category, no matter whether it is of particular behavioral relevance, represented in functionally specialized high-level visual areas during suppression? And in particular, are there differences between such high-level visual areas, e.g., ventral and dorsal stream areas, regarding the degree to which suppressed information is processed?

### PROCESSING OF EMOTIONALLY AND SOCIALLY RELEVANT INFORMATION UNDER INTEROCULAR SUPPRESSION

With regard to the question whether emotional information is processed during interocular suppression, results from fMRI studies consistently indicate enhanced processing of emotional facial expressions. Williams et al. (2004) presented either faces with neutral, happy, or fearful expressions to one eye and houses to the other eye. Stimuli were presented only for a short, fixed duration and the contrast and hue of the rivalrous images was manipulated so that just one image class was reliably perceived while the other image was suppressed. Activation in the fusiform face area (FFA) and parahippocampal place area (PPA) was increased for perceptually dominant versus suppressed faces and houses. In contrast, amygdala activation was increased in response to fearful versus neutral faces regardless of whether the face was dominant or suppressed, in line with the view that detection of emotional information proceeds automatically and does not require awareness (Vuilleumier, 2005). Similarly, during rivalry between a fearful face or a chair stimulus shown to one eye and a house stimulus (that was moving in order to ensure its dominance) to the other eye, activity in the amygdala was greater in response to suppressed fearful faces compared to chairs (Pasley et al., 2004). No such response difference was observed in ventral visual cortex in this study, from which the authors concluded that a high-level cortical representation is not required for the discrimination of certain behaviorally relevant stimuli in the amygdala. However, a more recent study in which fearful faces or houses were suppressed by moving checkerboards found stronger responses to fearful faces than to houses not only in the left amygdala, but also in left FFA (Troiani et al., 2012, but see Troiani and Schultz, 2013 for a failure to replicate these findings using CFS with high-contrast Mondrian-like masks).

It should be noted, however, that in the binocular rivalry studies mentioned above unawareness of the suppressed image was assessed either by using a one-back task that required participants to report repetitions of identical face or house stimuli (Williams et al., 2004), or by instructing participants to press a button if at any point they perceived anything else but the dominant house, checkerboard, or Mondrian-like stimulus (Pasley et al., 2004). As such methods do not reliably ensure objective unawareness of the suppressed stimuli, it cannot be ruled out that the observed response differences for suppressed faces might have been at least in part due to residual traces of stimulus awareness that went undetected by the tasks used.

In another fMRI study, CFS was used to render faces with fearful or neutral expressions invisible (Jiang and He, 2006). Here, a forced-choice task was used at least in behavioral pre- and post-scan sessions and showed that observers were unable to discriminate between suppressed intact and scrambled face stimuli, in addition to a subjective awareness assessment during fMRI scanning. Responses to invisible face stimuli in the FFA were strongly reduced relative to visible faces, but did not show differences between neutral and fearful expressions. In contrast, greater responses to fearful than to neutral faces were observed in the amygdala and in the superior temporal sulcus (see also Vizueta et al., 2012), a region previously implicated in the processing of changeable facial features such as expression or eye gaze (Haxby et al., 2000). In a subsequent EEG study from the same group (Jiang et al., 2009), the amplitude of the N170, a face-specific signal thought to reflect face processing in ventral occipitotemporal cortex, was not significantly different for fearful and neutral faces. In contrast, a later signal along the superior temporal sulcus was specific for fearful expressions. Further support for the notion that changeable facial features of particular social relevance might be processed without awareness along specialized neural pathways comes from a recent EEG study that found larger negative deflections at parietofrontal electrodes to suppressed faces with direct gaze compared to suppressed faces with averted gaze (Yokoyama et al., 2013). Although still exploratory, this finding is in line with behavioral evidence of unconscious processing of eye gaze under interocular suppression (Stein et al., 2011b, 2012; Xu et al., 2011; Chen and Yeh, 2012).

Together, neuroimaging studies of emotional face processing provide little evidence for processing of the category or the emotional information of suppressed object stimuli in high-level ventral visual areas such as the FFA. In contrast, both the amygdala and superior temporal sulcus show differential responses to suppressed fearful and neutral face stimuli. This is consistent with the notion of automatic processing of threat-signaling stimuli (Vuilleumier, 2005), which has been suggested to bypass the visual processing stages at which binocular conflict is resolved, possibly via subcortical pathways (LeDoux, 2000). Indeed, some fMRI studies provided indirect support for a role of subcortical pathways in driving amygdala activity to suppressed fearful faces by showing covarying activity between the amygdala and other visually responsive subcortical structures such as the superior colliculus (Pasley et al., 2004) and the pulvinar (Troiani et al., 2012; Troiani and Schultz, 2013). However, recent recordings from a depth electrode implanted in a patient’s amygdala revealed that responses to fearful faces rendered invisible through CFS occur only relatively late, after about 140 ms, and are driven by both low and high spatial frequencies in the facial stimuli (Willenbockel et al., 2012). These findings are inconsistent with the notion of a direct feedforward connection between the superior colliculus, pulvinar, and the amygdala, as this pathway is assumed to be particularly fast and to rely exclusively on low spatial frequency information. Similarly, a recent behavioral study has shown that privileged processing of threat-signaling visual stimuli does not rely on low spatial frequencies (Stein et al., 2014), again challenging the idea of a subcortical fast track for emotionally relevant visual information (for an in-depth discussion, see Pessoa and Adolphs, 2010). Clearly, more work is needed to pinpoint the neural networks underlying unconscious processing of emotionally charged and socially significant stimuli under rivalry suppression.

### OBJECT- AND CATEGORY-SPECIFIC PROCESSING IN HIGH-LEVEL AREAS OF THE DORSAL AND THE VENTRAL STREAM

**FIGURE 3:**
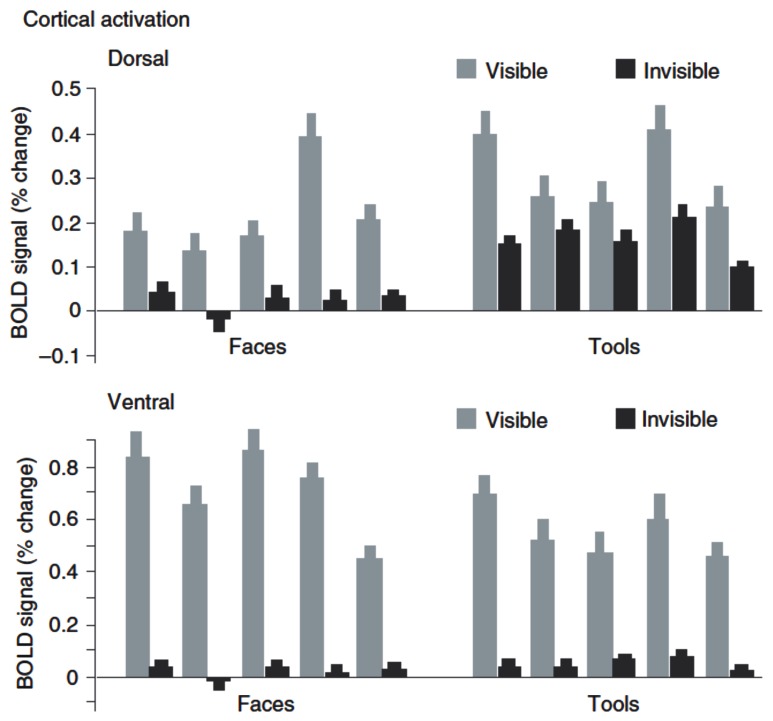
**fMRI-BOLD activation to visual stimuli suppressed by CFS.** Data from Fang and He (2005): Plotted are BOLD signals (% change) in dorsal and ventral stream to visible and invisible images of tools and faces. The ventral stream shows a strong reduction of activity under CFS, while the dorsal stream shows much less reduction when images are tools. Shown are data from five participants. Reproduced from Fang and He (2005; copyright 2005 Nature Publishing Group).

The other question that has been addressed by a number of recent neuroimaging studies is whether complex stimulus information regarding object identity or category can also survive suppression at early stages and be retained at advanced stages of visual processing. Importantly, just the absence of evidence for category-specific processing in specialized ventral visual areas during suppression (Pasley et al., 2004; Williams et al., 2004) cannot be taken as definite proof for the absence of such processing, as weak residual neural signals evoked by suppressed stimuli may have gone undetected by the neuroimaging methods used. Fang and He (2005) investigated neural responses to object stimuli suppressed by CFS in high-level areas pertaining to the ventral and dorsal streams of visual processing, respectively. Their stimuli included images of faces, which evoke mostly ventral activity, and images of tools, for which a dorsal preference has been shown. Similar to the abovementioned studies (Pasley et al., 2004; Williams et al., 2004), they did not observe any category-specific fMRI responses to invisible images of faces or tools in ventral visual areas. In contrast, dorsal regions did show responses to suppressed stimuli that were much less reduced in amplitude relative to visible stimuli (**Figure 3**), but exclusively for images of tools. The authors concluded that indeed substantial information from the suppressed eye could escape competitive interactions at early processing levels and reach dorsal visual areas, but not ventral areas. In line with previous evidence from lesion studies in humans and from animal studies (Milner and Goodale, 1995, 2006), they interpreted these findings as support for a fundamental specialization of the visual system into a dorsal *vision-for-action* stream and a ventral *vision-for-perception* stream. According to this theory, dorsal areas form action-relevant representations for selected types of visual objects, e.g., tools and other man-made manipulable objects, even in the absence of awareness, while there are no such representations in ventral visual areas.

**FIGURE 4:**
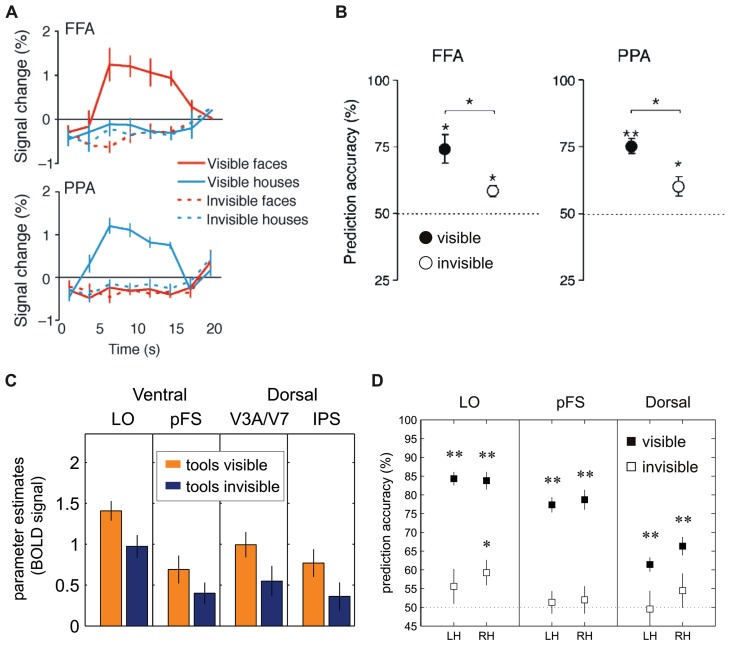
**Univariate and multivariate analysis of FMRI-BOLD activation to visual stimuli suppressed by CFS. (A)** Sterzer et al. (2008): The fusiform face area (FFA) and the parahippocampal place area (PPA) in inferior temporal cortex showed significantly reduced BOLD activity levels whenever images of faces or houses were rendered invisible. **(B)** Sterzer et al. (2008): Performance of support-vector-machine (SVM) classifiers for pairwise classification of face and house presentations from FFA and PPA. Filled circles: visible trials; open circles: invisible trials. **(C)** Hesselmann and Malach (2011): BOLD signals (parameter estimates in arbitrary units) to images of tools in dorsal and ventral visual areas show stream-invariant reduction whenever stimuli were rendered invisible (LO = lateral occipital area, pFS = posterior fusiform gyrus, IPS = intra-parietal sulcus). **(D)** Hesselmann and Malach (2011): Performance of SVM classifiers in left hemisphere (LH) and right hemisphere (RH) dorsal and ventral visual areas. Prediction accuracies in visible trials (filled squares) were significant in all regions-of-interest; in invisible trials (open squares), area LO showed classification performance significantly above chance level (*p < 0.01; **p < 0.001). **(A,B)** Reproduced from Sterzer et al. (2008; copyright 2008 Association for Research in Vision and Ophthalmology). **(C,D)** Modified from Hesselmann and Malach (2011; copyright 2011 Oxford University Press).

Findings from subsequent neuroimaging studies have provided partly diverging evidence that has questioned these conclusions. Using high-resolution fMRI study and multi-voxel pattern analysis to increase the sensitivity for distributed fMRI signals (Haynes and Rees, 2006; Norman et al., 2006) the fine-grained spatial activity patterns within the ventral areas FFA and PPA were shown to still contain information about the category of face and house stimuli even when the average BOLD signal was drastically reduced and stimuli were reliably suppressed from conscious perception, as evidenced by a rigorous objective awareness assessment during fMRI scanning (**Figures 4A,B**; Sterzer et al., 2008). Thus, the fine-grained spatial pattern of activity measured with fMRI in ventral visual areas encodes information about the identity of suppressed object stimuli. Similarly, face-specific electromagnetic responses to interocularly suppressed stimuli are reduced in amplitude but still present in the human ventral visual pathway (Sterzer et al., 2009a). These results are consistent with more general findings of high-level processing for stimuli outside awareness in other paradigms (e.g., see Kouider and Dehaene, 2007; Rees, 2007). Such unconscious high-level processing could provide a neural basis for how complex stimulus features contribute to the resolution of perceptual conflict even when suppressed (e.g., by high-level adaptation). The processing of suppressed stimuli, however, does not seem to extend to semantic information (i.e., semantic congruency between lexical units), as a recent EEG study found signals related to the semantic mismatch between two words (the N400) to be absent when participants could not discriminate the meaning of suppressed words (Kang et al., 2011).

A recently published study also questioned the distinction between dorsal and ventral visual areas in the processing of suppressed object stimuli (Hesselmann and Malach, 2011). In this study participants had to detect, during CFS, images of tools. Despite their substantial difference in connectivity and neuroanatomical specialization, both ventral and dorsal stream areas revealed a similarly tight link to perceptual awareness, that is, strong fMRI signals for visible tools but a significant reduction of activity in the invisible condition (**Figure 4C**). In other words, this study failed to replicate the previous finding (Fang and He, 2005) that specifically dorsal areas contain representations of manipulable objects during binocular rivalry suppression. Another interesting observation from this study is that CFS did not lead to a complete abolition of category-specific activity in response to invisible stimuli, as object category could still be decoded from fMRI signal patterns in lateral occipital cortex with multi-voxel pattern analyses (**Figure 4D**), in line with the abovementioned previous work (Sterzer et al., 2008). The divergent findings between the studies by Fang and He (2005) and Hesselmann and Malach (2011) may be explained by differences in study design, especially with respect to the behavioral assessment of unawareness. In the experiments of Fang and He (2005), participants were asked to report whether they perceived any shape or object after prolonged blocks of fMRI scanning while their task was to detect occasional size changes of the fixation point. Only a subset of participants performed a trial-wise forced-choice task in separate control experiments (“offline”) to establish objective absence of awareness. In contrast, Hesselmann and Malach (2011) used a trial-by-trial forced-choice task during the main fMRI experiment (“online”), which constitutes a more direct and arguably more sensitive test for visibility. As high-order visual areas specialized on object processing are very sensitive even to poorly visible low contrast images or object parts (Avidan et al., 2002; Lerner et al., 2002), it cannot be excluded, because of the comparably insensitive assessment of unawareness, that responses to invisible tools in the study by Fang and He (2005) were at least in part due to occasional traces of residual target visibility. However, against this argument of residual but unreported visibility seems to speak the fact that only dorsal but not ventral stream areas showed preserved activity under CFS.

In a further study that focused on the relationship between report type, subjective versus objective, and fMRI responses to face or tool stimuli during CFS, Hesselmann et al. (2011) replicated their previous finding of similar fMRI signal reductions in both ventral and dorsal visual areas when stimuli were invisible. In addition, they showed a dissociation between type of report and low- vs. high-level visual areas: Activity in high-level visual areas was enhanced when subjects reported higher levels of subjective visibility, even when objective performance was constant. In contrast, with constant subjective performance, these areas showed no activity differences between trials with objectively correct or incorrect responses. On the other hand, objective behavioral performance was linked to the accuracy of multivariate pattern classification mainly in early visual areas, thus suggesting that subjective and objective reports tap cortical signals of different location and amplitude within the visual cortex (Hesselmann et al., 2011).

In summary, neuroimaging studies investigating the processing of visual information during interocular suppression have shown repeatedly that object- or category-specific neural activity in high-level visual areas of the ventral stream is strongly reduced, but can be retrieved when sufficiently sensitive methods of data analysis are used, such as multi-voxel pattern analysis of fMRI data. It will be an important challenge for future research to determine to what degree such residual traces of object-related neural activity are relevant behaviorally, e.g., in that they influence the access of object information to awareness (see below). Research into a putative dissociation of ventral and dorsal stream areas in the processing of object information has not provided conclusive results yet. Possibly, dorsal areas are more sensitive than ventral areas to the presence of weak or noisy information, but responses in dorsal and ventral areas seem to be reduced to a similar degree when object stimuli are fully and objectively suppressed from awareness.

## CURRENT CHALLENGES AND FUTURE DIRECTIONS

As outlined in this review, neuroimaging studies of interocular suppression have provided important new insights into unconscious visual information processing, but also generated new controversies. When trying to draw a coherent picture of the neural events that are related to the processing of visual information under interocular suppression, one of the major challenges at the current stage is the heterogeneity of findings. This is the case both when we ask whether a given neural structure is involved in processing of suppressed stimuli at all, but also when it comes to the question of feature- or category-specific processing in the absence of awareness. At least some of the inconsistencies between studies may be related to differences in the depth of interocular suppression. There are in principle two scenarios that could account for heterogeneous findings on the basis of suppression depth: In the first scenario, suppression is not deep enough and the stimulus breaks through and is partially or even fully visible, at least from time to time. If awareness is not assessed stringently on a trial-to-trial basis, this could result in false-positive findings and the erroneous conclusion that neural processing is preserved in the absence of awareness in cases where in fact it is not. In the second scenario, suppression could be too deep, thereby fully abolishing neural responses that could in principle still occur in the absence of awareness. Such a scenario could result in false-negative conclusions. Future studies should aim at avoiding both these scenarios by taking great care in defining those conditions under which neural processing of a stimulus is not unnecessarily deadened despite reliable suppression from awareness. Promising approaches could be to systematically vary the properties of the mask and/or the target stimulus that are most relevant for the depth of interocular suppression, such as stimulus contrast and spatial frequency (Yang and Blake, 2012), or to adjust the suppression threshold individually to a point where stimulus power is as high as possible but as low as necessary (for suppression to work). As discussed above, careful assessment and documentation of unawareness will be of key importance to any study concerned with neural processing under interocular suppression, as this will help the interpretation of each study’s findings as well as the comparison of findings between studies.

A point that has received little attention to date concerns the functional relevance of neural signals that are recorded under conditions of interocular suppression. Are preserved neural responses to suppressed stimuli relevant for behavior, or could they be entirely irrelevant and thus just “epiphenomenal?” To assess the functional relevance of unconscious visual information processing under interocular suppression, many studies have measured how invisible stimuli modulate behavioral responses to a succeeding visible stimulus, adopting priming, adaptation aftereffects, or attentional cueing paradigms (Moradi et al., 2005; Jiang et al., 2006; Almeida et al., 2008, 2013; Stein and Sterzer, 2011; Anderson et al., 2012; Faivre et al., 2012). By using stimuli and tasks of different complexity, such behavioral methods have been used to indirectly infer unconscious neural processing at different levels of the visual hierarchy. What is lacking to date however, are neuroimaging studies that use such behavioral measures concurrently to directly establish the functional relevance of brain signals measured under interocular suppression.

One limitation of the behavioral measures of unconscious processing discussed so far is that they assess effects of suppressed stimuli *after* they have been presented outside awareness. They are therefore limited by the potentially short-lived nature of unconscious effects (Greenwald et al., 1996) and constrained by specific task requirements. So far, only few studies have measured behavioral effects of interocularly suppressed stimuli on-line, that is, *during* the presentation of stimuli outside awareness. One way to do so is to monitor motor behavior related to the invisible stimulus during presentation. A recent study analyzed grasping movements to stimuli that were suppressed from awareness by CFS (Roseboom and Arnold, 2011). The authors found that participants learned to adjust the orientation of their hand to the stimulus orientation over the course of the experiment. In contrast, applying a more rigorous control of stimulus visibility across sessions, another study (Ludwig et al., 2013) failed to find evidence for the use of unconscious stimulus information by the visuomotor system: Participants neither learned to adjust the size of their grip aperture nor the orientation of their hand to invisible stimuli. Thus, whether grasping movements are indeed a useful way of measuring the behavioral effects of unconscious visual processing under interocular suppression awaits further clarification. Possibly, the monitoring of eye movements may prove a more useful approach: Using eye movements as a behavioral response measure, it was recently demonstrated that observers spend more time looking at suppressed stimuli despite being unable to correctly guess the stimulus location in a manual forced-choice task (Rothkirch et al., 2012). Eye movement recordings thus seem to be a promising technique to determine the functional relevance of neural signals recorded during interocular suppression (see also Spering et al., 2011; Spering and Carrasco, 2012, for a dissociation of eye movements and reported perception).

Another technique that has recently become very popular is “breaking-CFS” (b-CFS), which measures the time it takes until a stimulus breaks into awareness after initial suppression through CFS, thus supposedly indicating the strength of neural processing while the stimulus is still suppressed (Jiang et al., 2007). However, whether b-CFS actually reflects unconscious processing is currently a matter of debate (Stein et al., 2011a; Stein and Sterzer, 2014). Neuroimaging studies could help resolving this debate by demonstrating a tight coupling between neural responses to the initially invisible stimulus and the duration of perceptual suppression (Yamashiro et al., 2013). If brain signals during full suppression predicted subsequent breakthrough into awareness on a trial-by-trial basis, this would provide direct evidence for the functional relevance of unconscious neural processing in mediating access to awareness.

Since evidence for functional relevance of neural signals in response to interoculary suppressed stimuli is still sparse, further research is warranted to provide a better understanding of how such unconscious visual information can modulate behavior, and which neural processes might mediate such effects. This is a challenging task, as it requires observers to be unaware of the association between a suppressed stimulus and their own behavior. It also seems crucial to learn more about which behavioral measures are best suited to study behavioral responses under interocular suppression. For instance, continuous behavioral measures might capture neural activity related to suppressed stimuli that is not reflected in discrete measures, such as manual button presses (Fahle et al., 2011; Naber et al., 2011). It remains an intriguing challenge for future research to establish experimental approaches that allow us to explore the functional relevance of neural signals measured in response to visual stimuli during interocular suppression.

## Conflict of Interest Statement

The authors declare that the research was conducted in the absence of any commercial or financial relationships that could be construed as a potential conflict of interest.
